# Description of Residual Stresses in Autofrettaged Open-Ended Cylinders Made of High-Strength Steel

**DOI:** 10.3390/ma13132940

**Published:** 2020-06-30

**Authors:** Sergei Alexandrov, Elena Lyamina, Jong-Ning Aoh, Yeau-Ren Jeng

**Affiliations:** 1 Division of Computational Mathematics and Engineering, Institute for Computational Science, Ton Duc Thang University, Ho Chi Minh City 700000, Vietnam; sergeialexandrov@tdtu.edu.vn; 2 Faculty of Civil Engineering, Ton Duc Thang University, Ho Chi Minh City 700000, Vietnam; 3 Ishlinsky Institute for Problems in Mechanics RAS, Moscow 119526, Russia; lyamina@inbox.ru; 4 Department of Mechanical Engineering, National Chung Cheng University, Chiayi 62102, Taiwan; imejna@ccu.edu.tw; 5 Advanced Institute of Manufacturing with High-Tech Innovations (AIM-HI), National Chung Cheng University, Chiayi 62102, Taiwan; 6 Department of Biomedical Engineering, National Cheng Kung University, Tainan City 70101, Taiwan

**Keywords:** residual stress, open-ended cylinder, autofrettage, Bauschinger effect

## Abstract

The elastic range in loading–unloading processes is often reduced with a Bauschinger effect. This material property may have a high impact on residual stresses and, as a result, on the performance of autofrettaged cylinders under service conditions. The objective of the present paper is to demonstrate this impact using a material model that accounts for the response of typical high-strength steel. The solution is semi-analytic and, therefore, can be used for fast and accurate analysis of the process of autofrettage. A numerical example illustrates the general solution. This example shows that the Bauschinger effect has a significant impact on the residual circumferential stress in the vicinity of the inner radius of the cylinder. This stress is the most significant quantity of autofrettaged cylinders. Therefore, the main result obtained suggests that even a moderate Bauschinger effect should be taken into account in analyses of the process of autofrettage.

## 1. Introduction

The elastic range in loading–unloading processes is often reduced with a Bauschinger effect. This material property has a substantial impact on the distribution of stresses in autofrettaged cylinders. Many types of high-strength steel show little or no forward hardening but a strong Bauschinger effect [1,2,3,4]. Most of the available solutions for such materials do not require the yield locus but only two points of this locus [5,6,7,8]. Analysis of the autofrettage of open-ended cylinders made of high-strength steel under plane stress conditions needs a more general model that predicts the perfectly plastic behavior of the material at loading and accounts for the Bauschinger effect at unloading. Such a material model has been proposed in [9]. This model is adopted in the present paper.

The autofrettage technology induces a favorable residual stress field for increasing the load capacity of high-pressure vessels. Several processes are used to autofrettage cylindrical pressure vessels and disks, such as hydrostatic, swage, rotational, and thermal autofrettage [10,11,12,13]. The constitutive equations adopted may significantly affect theoretical predictions of residual stress fields after the autofrettage process. Therefore, the theory of autofrettage has been intensively discussed in the literature. It is sufficient to mention pioneering works [10,14] and a very recent comprehensive review [15]. None of the solutions included in this review accounts for the specific features of the Bauschinger effect in high-strength steels. An influence of plastic anisotropy on the distribution of residual stresses and strains in open-ended, autofrettaged cylinders has been investigated in [16]. Experimental studies for materials that reveal the Bauschinger effect have been reported in [17,18,19]. The objective of the present research is rather to develop a simple theory of autofrettage of open-ended cylinders made of high-strength steel based on the model [9]. In particular, a semi-analytic solution for the stage of loading is available in [20]. The stress solution for the stage of unloading requires solving an ordinary differential equation and transcendental equations numerically. It is shown that the impact of the Bauschinger effect on the distribution of the residual circumferential stress in the vicinity of the inner radius of the cylinder is significant. This stress is the most significant quantity of autofrettaged cylinders. Therefore, the main result obtained suggests that even a moderate Bauschinger effect should be taken into account in analyses of the process of autofrettage. 

## 2. Statement of the Problem and Basic Equations

**Figure 1:**
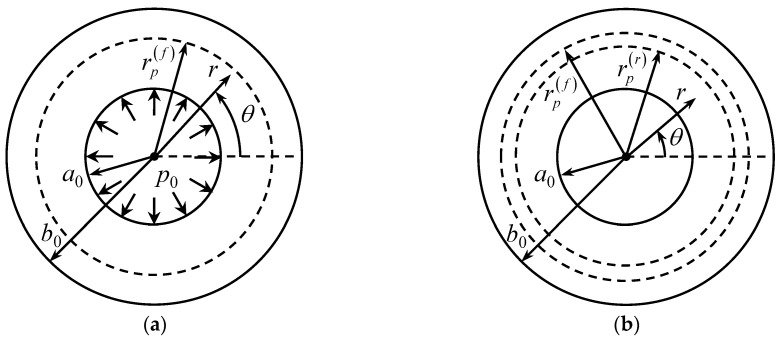
Illustration of the boundary value problem: (**a**) loading; (**b**) unloading.

Consider a long open-ended cylinder of initial yield stress $\sigma_{0}$, Young’s modulus *E*, Poisson’s ratio υ, outer radius $b_{0}$, and inner radius $a_{0}$. The cylinder is subject to uniform pressure $p_{0}$ over its inner radius, followed by unloading. The pressure is sufficient to yield the material to an intermediate radius $r_{p}^{\left(f\right)}$ at loading and $r_{p}^{\left(r\right)}$ in reversed flow. The outer radius of the cylinder is stress-free. Figure 1 illustrates the boundary value problem. It is natural to use the cylindrical coordinate system (r, θ, z), as shown in this figure. The solution is independent of θ, and the principal stress trajectories coincide with the coordinate curves of this coordinate system. The normal stresses referred to the cylindrical coordinate system, which are the principal stresses, are denoted as $\sigma_{r}$, $\sigma_{\theta }$ and $\sigma_{z}$. Moreover, it is assumed that the state of stress is plane stress such that $\sigma_{z}=0$.

**Figure 2:**
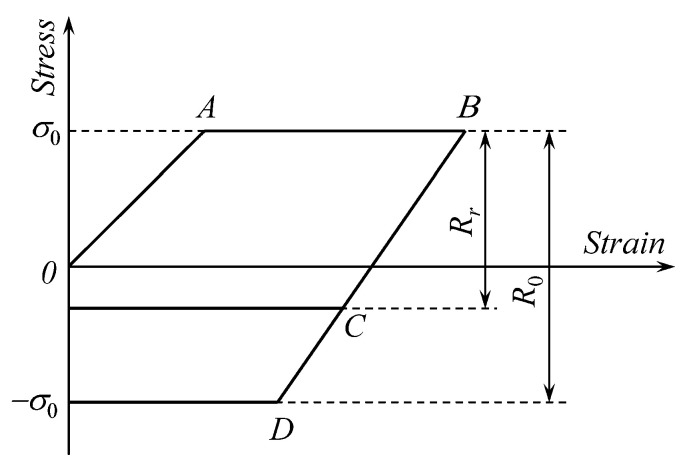
Geometric illustration of the Bauschinger effect considered in the present paper.

A general feature of the class of materials considered in the present paper is that there is little or no forward hardening, but a significant Bauschinger effect. This feature of constitutive material behavior is illustrated in Figure 2 for one-dimensional loading. Forward deformation is represented by the line *OAB*, where *OA* corresponds to elastic deformation and *AB* to elastic/plastic deformation. Line *BD* represents the elastic unloading in materials with no Bauschinger effect. In this case, the elastic range is *R*_0_. Line *BC* represents the elastic unloading in materials that reveal a Bauschinger effect. In this case, the elastic range becomes *R_r_* where *R_r_* < *R*_0_.

Taking into account the discussion above, the constitutive equations at loading constitute Hooke’s law, a yield criterion of perfect plasticity under plane stress conditions and its associated flow rule. In particular, the von Mises yield criterion under plane stress conditions takes the form

(1) $$\sigma_{r}^{2}+\sigma_{\theta }^{2}-\sigma_{\theta }\sigma_{r}=\sigma_{0}^{2}.$$

Let $\varepsilon_{r}^{p}$, $\varepsilon_{\theta }^{p}$ and $\varepsilon_{z}^{p}$ be the plastic strain components referred to the cylindrical coordinate system. Then, the plastic flow rule is

(2) $${\dot{\varepsilon }}_{r}^{p}=\lambda \left(\sigma_{r}-\sigma \right),\;{\dot{\varepsilon }}_{\theta }^{p}=\lambda \left(\sigma_{\theta }-\sigma \right),\;{\dot{\varepsilon }}_{z}^{p}=-\lambda \sigma .$$

Here, σ is the hydrostatic stress, $\sigma =\left(\sigma_{r}+\sigma_{\theta }\right)/3$, λ is a non-negative multiplier, and the superimposed dot denotes the derivative with respect to a time-like parameter, *t*. The elastic strain components, $\varepsilon_{r}^{e}$, $\varepsilon_{\theta }^{e}$ and $\varepsilon_{z}^{e}$, are connected to the stress components as

(3) $$\varepsilon_{r}^{e}=\frac{\sigma_{r}-\nu \sigma_{\theta }}{E},\;\varepsilon_{\theta }^{e}=\frac{\sigma_{\theta }-\nu \sigma_{r}}{E},\;\varepsilon_{z}^{e}=-\frac{\nu \left(\sigma_{r}+\sigma_{\theta }\right)}{E}.$$

The components of the total strain tensor are

(4) $$\varepsilon_{r}^{}=\varepsilon_{r}^{e}+\varepsilon_{r}^{p},\;\varepsilon_{\theta }^{}=\varepsilon_{\theta }^{e}+\varepsilon_{\theta }^{p}\;\mathrm{and}\;\varepsilon_{z}^{}=\varepsilon_{z}^{e}+\varepsilon_{z}^{p}.$$

It is assumed that the forward plastic strain components affect the reversed yield criterion. In particular, according to Prager’s law [21], the reversed yield criterion under plane stress conditions is

(5) $$\frac{3}{2}\left[{\left(\sigma_{r}-\sigma -C\varepsilon_{r}^{p}\right)}^{2}+{\left(\sigma_{\theta }-\sigma -C\varepsilon_{\theta }^{p}\right)}^{2}+{\left(\sigma +C\varepsilon_{z}^{p}\right)}^{2}\right]=\sigma_{0}^{2}$$

where *C* is a material constant. The plastic flow rule associated with the yield criterion (5) is

(6) $${\dot{\varepsilon }}_{r}^{p}=\lambda_{1}\left(\sigma_{r}-\sigma -C\varepsilon_{r}^{pf}\right),\;{\dot{\varepsilon }}_{\theta }^{p}=\lambda_{1}\left(\sigma_{\theta }-\sigma -C\varepsilon_{\theta }^{pf}\right),\;{\dot{\varepsilon }}_{z}^{p}=-\lambda_{1}\left(\sigma +C\varepsilon_{z}^{pf}\right).$$

Here, and in the solution for the stage of unloading, the superscript *f* denotes the forward strain. 

The constitutive equations above should be supplemented with the only non-trivial equilibrium equation:

(7) $$\frac{\partial \sigma_{r}}{\partial r}+\frac{\sigma_{r}-\sigma_{\theta }}{r}=0.$$

It is convenient to use the following dimensionless quantities:

(8) $$a=\frac{a_{0}}{b_{0}},\;\rho =\frac{r}{b_{0}},\;k=\frac{\sigma_{0}}{E},\;p=\frac{p_{0}}{\sigma_{0}}.$$

In particular, Equation (7) becomes

(9) $$\frac{\partial \sigma_{r}}{\partial \rho }+\frac{\sigma_{r}-\sigma_{\theta }}{\rho }=0.$$

The boundary conditions at the stage of forward loading are

(10) $$\frac{\sigma_{r}}{\sigma_{0}}=-p\;\mathrm{for}\;\rho =a$$

and

(11) $$\frac{\sigma_{r}}{\sigma_{0}}=0\;\mathrm{for}\;\rho =1.$$

The boundary conditions at the stage of unloading are

(12) $$\frac{\Delta \sigma_{r}}{\sigma_{0}}=p_{f}\;\mathrm{for}\;\rho =a$$

and

(13) $$\frac{\Delta \sigma_{r}}{\sigma_{0}}=0\;\mathrm{for}\;\rho =1.$$

Here $\Delta \sigma_{r}$ is the increment of the radial stress in the course of unloading and $p_{f}$ is the value of *p* at the end of loading.

The material model above has been proposed in [9].

## 3. Solution at Loading

A solution at loading has been proposed in [20]. This solution is outlined in this section to supply the equations that are necessary for determining the distribution of residual stresses after unloading. In what follows, $p_{f}$ will denote the value of *p* at the end of loading. 

The general stress solution in the elastic region is well known [10]. This solution, satisfying the boundary condition (11), is represented as

(14) $$\frac{\sigma_{r}}{\sigma_{0}}=A\left(\frac{1}{\rho^{2}}-1\right)\;\mathrm{and}\;\frac{\sigma_{\theta }}{\sigma_{0}}=-A\left(\frac{1}{\rho^{2}}+1\right).$$

Here *A* is a function of *p*. The strain solution is immediate from (1), (3), (8), and (14). As a result,

(15) $$\frac{\varepsilon_{r}^{e}}{k}=A\left[\frac{\left(1+\nu \right)}{\rho^{2}}-1+\nu \right],\;\frac{\varepsilon_{\theta }^{e}}{k}=-A\left[\frac{\left(1+\nu \right)}{\rho^{2}}+1-\nu \right],\;\frac{\varepsilon_{z}^{e}}{k}=2\nu A.$$

The yield criterion (1) is satisfied by the following standard substitution:

(16) $$\frac{\sigma_{r}}{\sigma_{0}}=-\frac{2\sin\psi }{\sqrt{3}}\;\mathrm{and}\;\frac{\sigma_{\theta }}{\sigma_{0}}=-\frac{\sin\psi }{\sqrt{3}}-\cos\psi .$$

Here, ψ is a new unknown function of ρ. Equations (9) and (16) combine to give

(17) $$2\rho \cos\psi \frac{\partial \psi }{\partial \rho }=\sqrt{3}\cos\psi -\sin\psi .$$

The distribution of the principal stresses is given by (14) in the range $\rho_{c}\leq \rho \leq 1$ and by (16) in the range $a\leq \rho \leq \rho_{c}$. Here, $\rho_{c}$ is the dimensionless radius of the elastic/plastic interface. Then, using (16), one can rewrite the boundary condition (10) as

(18) $$\frac{2}{\sqrt{3}}\sin\psi_{a}=p$$

where $\psi_{a}$ is the value of ψ at ρ=a. The solution of Equation (17) satisfying the boundary condition (18) is

(19) $$\rho =a\exp\left[\frac{\sqrt{3}}{2}\left(\psi -\psi_{a}\right)\right]\sqrt{\frac{\sin\left(\psi_{a}-\pi /3\right)}{\sin\left(\psi -\pi /3\right)}}.$$

Equations (16) and (19) supply the dependence of the stress components on the dimensionless radius in parametric form. 

It is seen from (18) that $p_{f}\leq \sqrt{3}/2$ and $\psi_{a}$ is a monotonic function of *p* in the range $0\leq p\leq p_{f}$. Therefore, it is possible to assume with no loss of generality that $t\equiv \psi_{a}$ (*t* has been introduced after Equation (2)). It is seen from (4) that ${\dot{\varepsilon }}_{\theta }={\dot{\varepsilon }}_{\theta }^{e}+{\dot{\varepsilon }}_{\theta }^{p}$. The dependence of ${\dot{\varepsilon }}_{\theta }$ on ψ in the plastic region is given by

(20) $$\begin{array}{c} \frac{{\dot{\varepsilon }}_{\theta }}{k}=\frac{{\dot{\varepsilon }}_{c}}{k}\exp\left[\sqrt{3}\left(\psi_{c}-\psi \right)\right]+\frac{\cos\psi_{a}}{\sqrt{3}\left(\sqrt{3}\cos\psi_{a}-\sin\psi_{a}\right)}\times \\ \int_{\psi_{c}}^{\psi }\frac{\left[\left(1-2\nu \right)\left(\sqrt{3}\sin2\mu -\cos2\mu \right)-2\left(2-\nu \right)\right]}{\cos\mu }\exp\left[\sqrt{3}\left(\mu -\psi \right)\right]d\mu . \end{array}$$

Here, μ is a dummy variable of integration and ${\dot{\varepsilon }}_{c}$ is the value of ${\dot{\varepsilon }}_{\theta }$ at $\rho =\rho_{c}$. The quantities ${\dot{\varepsilon }}_{c}$, $\psi_{c}$, and $\rho_{c}$ are functions of $\psi_{a}$. Choose an arbitrary value of ρ in the range $a\leq \rho <\rho_{c}$. This value of ρ will denoted as $\rho_{i}$. At $\rho =\rho_{i}$, ψ is a function of $\psi_{a}$, as follows from (19). One can eliminate ψ in (20) using this function. Then, the right-hand side of (20) becomes a function of $\psi_{a}$, ${\dot{\varepsilon }}_{\theta }/k=E_{\theta }\left(\psi_{a}\right)$. The resulting equation can be immediately integrated to give the value of the total circumferential strain at $\rho =\rho_{i}$ at the end of loading as

(21) $$\frac{\varepsilon_{\theta }}{k}=\int_{\psi_{i}}^{\psi_{f}}E_{\theta }\left(\psi_{a}\right)d\psi_{a}+\frac{\varepsilon_{\theta }^{\left(i\right)}}{k}.$$

Here, $\psi_{i}$ is the value of $\psi_{a}$, at which $\rho_{c}=\rho_{i}$, $\psi_{f}$ is determined from (18) at $p=p_{f}$, and $\varepsilon_{\theta }^{\left(i\right)}$ is the elastic circumferential strain at the elastic/plastic boundary at the instant when $\rho_{c}=\rho_{i}$. The value of $\varepsilon_{\theta }^{\left(i\right)}$ is found from (15). The elastic portion of the circumferential strain is determined from (3) and (16). Having found the elastic portion, the plastic portion of the circumferential strain is immediate from (4) and (21).

The plastic portions of the radial and axial strains can be found in a similar manner. In particular,

(22) $${\dot{\varepsilon }}_{r}^{p}={\dot{\varepsilon }}_{\theta }^{p}\frac{\sin\left(\psi -\pi /6\right)}{\cos\psi }\;\mathrm{and}\;{\dot{\varepsilon }}_{z}^{p}=-{\dot{\varepsilon }}_{\theta }^{p}\frac{\sin\left(\psi +\pi /6\right)}{\cos\psi }.$$

Since $\varepsilon_{r}^{p}=\varepsilon_{z}^{p}=0$ at $\psi_{a}=\psi_{i}$, one can rewrite (22) as

(23) $$\varepsilon_{r}^{p}=\int_{\psi_{i}}^{\psi_{f}}{\dot{\varepsilon }}_{\theta }^{p}\frac{\sin\left(\psi -\pi /6\right)}{\cos\psi }d\psi_{a}\;\mathrm{and}\;\varepsilon_{z}^{p}=-\int_{\psi_{i}}^{\psi_{f}}{\dot{\varepsilon }}_{\theta }^{p}\frac{\sin\left(\psi +\pi /6\right)}{\cos\psi }d\psi_{a}.$$

These equations supply the forward plastic strains $\varepsilon_{r}^{p}$ and $\varepsilon_{z}^{p}$ at $\rho =\rho_{i}$ and $\psi_{a}=\psi_{f}$. Using integration by parts, one transforms the equations in (23) to

(24) $$\begin{array}{c} \varepsilon_{r}^{p}=\frac{\sin\left(\psi_{f}-\pi /6\right)}{\cos\psi_{f}}{\varepsilon_{\theta }^{p}|}_{\psi_{a}=\psi_{f}}-\frac{\sqrt{3}}{2}\int_{\psi_{i}}^{\psi_{f}}\frac{\varepsilon_{\theta }^{p}}{\cos^{2}\psi }\frac{d\psi }{d\psi_{a}}d\psi_{a},\\ \varepsilon_{z}^{p}=-\frac{\sin\left(\psi_{f}+\pi /6\right)}{\cos\psi_{f}}{\varepsilon_{\theta }^{p}|}_{\psi_{a}=\psi_{f}}+\frac{\sqrt{3}}{2}\int_{\psi_{i}}^{\psi_{f}}\frac{\varepsilon_{\theta }^{p}}{\cos^{2}\psi }\frac{d\psi }{d\psi_{a}}d\psi_{a}. \end{array}$$

It has been taken into account here that $\varepsilon_{\theta }^{p}=0$ at $\psi_{a}=\psi_{i}$. At $\rho =\rho_{i}$, one can eliminate ψ in the integrands in (24) using (19). The plastic portion of the circumferential strain is immediate from (21) and Hooke’s law. It remains to determine the derivative $d\psi /d\psi_{a}$ at $\rho =\rho_{i}$. Since $d\left(\rho_{i}^{2}\right)/d\psi_{a}=0$, it follows from (19) that

(25) $$\frac{d\psi }{d\psi_{a}}=\frac{\sin\left(\psi -\pi /3\right)\left[\sqrt{3}\sin\left(\psi_{a}-\pi /3\right)-\cos\left(\psi_{a}-\pi /3\right)\right]}{\sin\left(\psi_{a}-\pi /3\right)\left[\sqrt{3}\sin\left(\psi -\pi /3\right)-\cos\left(\psi -\pi /3\right)\right]}.$$

Using (19) and (25), one can express the derivative $d\psi /d\psi_{a}$ as a function of $\psi_{a}$. Then, the integrals in (24) can be evaluated.

A full description of this method of solution, including the system of equations for determining ${\dot{\varepsilon }}_{c}$, $\psi_{c}$, $\psi_{a}$, $\rho_{c}$, and *A* as functions of *p*, is provided in [20]. In what follows, it is assumed that the solution at loading is available, including the plastic strains involved in (5) and (6).

It is worthy of note that all strains are proportional to *k*. This is seen from (15), (21), and (24). Therefore, the value of *k* is immaterial for theoretical solutions. In particular, assume that the solution for a cylinder of a given material is available. Then, simple scaling of this solution provides the solutions for similar cylinders of material with the same Poisson’s ratio but any value of *k*. For this reason, the solution in the next section will be derived in terms of $\xi_{r}=\varepsilon_{r}/k$, $\xi_{\theta }=\varepsilon_{\theta }/k$ and $\xi_{z}=\varepsilon_{z}/k$ instead of the strain components.

## 4. Stress Solution at Unloading

Using the general stress solution given in [10], one can determine the increments of the principal stresses in the following form:

(26) $$\frac{\Delta \sigma_{r}}{\sigma_{0}}=\frac{\Delta A}{\rho^{2}}+\Delta B\;\mathrm{and}\;\frac{\Delta \sigma_{\theta }}{\sigma_{0}}=-\frac{\Delta A}{\rho^{2}}+\Delta B$$

where ΔA and ΔB are new constants of integration. It follows from (12), (13) and (26) that

(27) $$\Delta A=\frac{p_{f}a^{2}}{1-a^{2}}\;\mathrm{and}\;\Delta B=\frac{p_{f}a^{2}}{a^{2}-1}.$$

Substituting (27) into (26) gives

(28) $$\frac{\Delta \sigma_{r}}{\sigma_{0}}=\frac{p_{f}a^{2}\left(1-\rho^{2}\right)}{\left(1-a^{2}\right)\rho^{2}}\;\mathrm{and}\;\frac{\Delta \sigma_{\theta }}{\sigma_{0}}=-\frac{p_{f}a^{2}\left(1+\rho^{2}\right)}{\left(1-a^{2}\right)\rho^{2}}.$$

The yield criterion (5) can be rewritten as

(29) $$\begin{array}{c} {\left[\frac{\left(2\sigma_{r}^{f}-\sigma_{\theta }^{f}\right)}{\sigma_{0}}+\frac{\left(2\Delta \sigma_{r}^{}-\Delta \sigma_{\theta }^{}\right)}{\sigma_{0}}-3c\xi_{r}^{pf}\right]}^{2}+{\left[\frac{\left(2\sigma_{\theta }^{f}-\sigma_{r}^{f}\right)}{\sigma_{0}}+\frac{\left(2\Delta \sigma_{\theta }^{}-\Delta \sigma_{r}^{}\right)}{\sigma_{0}}-3c\xi_{\theta }^{pf}\right]}^{2}+\\ {\left[\frac{\left(\sigma_{r}^{f}+\sigma_{\theta }^{f}\right)}{\sigma_{0}}+\frac{\left(\Delta \sigma_{r}^{}+\Delta \sigma_{\theta }^{}\right)}{\sigma_{0}}+3c\xi_{z}^{pf}\right]}^{2}\leq 6 \end{array}$$

where c=C/E. The solution (28) is valid if this inequality is not violated in the range a≤ρ≤1. The solution at loading and (28) show that it is sufficient to check (29) at ρ=a. It is evident from (10) and (12) that

(30) $$\sigma_{r}+\Delta \sigma_{r}=0$$

at ρ=a. Using (16), (18) and (28) one can get

(31) $$\frac{\sigma_{\theta }+\Delta \sigma_{\theta }}{\sigma_{0}}=\sqrt{1-\frac{3}{4}p_{f}^{2}}-\frac{\left(3-a^{2}\right)}{2\left(1-a^{2}\right)}p_{f}=q_{f}.$$

Substituting (30) and (31) into (29) one arrives at

(32) $$q_{f}^{2}-3cq_{f}\xi_{\theta }^{pf}+\frac{3}{2}c^{2}\left[{\left(\xi_{r}^{pf}\right)}^{2}+{\left(\xi_{\theta }^{pf}\right)}^{2}+{\left(\xi_{z}^{pf}\right)}^{2}\right]\leq 1.$$

The forward plastic strains are understood to be calculated at ρ=a. The equation $\xi_{r}^{pf}+\xi_{\theta }^{pf}+\xi_{z}^{pf}=0$ that follows from the equation $\varepsilon_{r}^{pf}+\varepsilon_{\theta }^{pf}+\varepsilon_{z}^{pf}=0$ has been used to derive (32). The equation $\varepsilon_{r}^{pf}+\varepsilon_{\theta }^{pf}+\varepsilon_{z}^{pf}=0$ follows immediately from (2). 

Equations (31) and (32) combine to supply the equation for determining the maximum possible value of $p_{f}$ at which the process of unloading is purely elastic. This value of $p_{f}$ is denoted as $p_{f}^{e}$. It is worthy of note that the values of $\xi_{r}^{pf}$, $\xi_{\theta }^{pf}$ and $\xi_{z}^{pf}$ involved in (32) depend on $p_{f}$.

In what follows, it is assumed that $p_{f}>p_{f}^{e}$. Therefore, a reversed plastic region occurs in the course of unloading. The radius of this region is denoted as $r_{p}^{\left(r\right)}$ (Figure 1) and its dimensionless representation as $\rho_{s}=r_{p}^{\left(r\right)}/b_{0}$. The solution (26) is valid in the region $\rho_{s}\leq \rho \leq 1$. However, ΔA and ΔB are not determined from (27). The yield criterion (5) is valid in the region $a\leq \rho \leq \rho_{s}$. This criterion is satisfied by the substitution

(33) $$\frac{T_{r}}{\sigma_{0}}=\frac{2\sin\gamma }{\sqrt{3}}\;\mathrm{and}\;\frac{T_{\theta }}{\sigma_{0}}=\frac{\sin\gamma }{\sqrt{3}}+\cos\gamma$$

where

(34) $$\frac{T_{r}}{\sigma_{0}}=\frac{\sigma_{r}}{\sigma_{0}}-c\xi_{r}^{pf}+c\xi_{z}^{pf}\;\mathrm{and}\;\frac{T_{\theta }}{\sigma_{0}}=\frac{\sigma_{\theta }}{\sigma_{0}}-c\xi_{\theta }^{pf}+c\xi_{z}^{pf}.$$

Furthermore, γ is a new unknown function of ρ. Since ψ is a known monotonic function of ρ in the region $a\leq \rho \leq \rho_{s}$, Equation (9) can be rewritten as

(35) $$\frac{\partial \sigma_{r}}{\partial \psi }\frac{\partial \psi }{\partial \rho }+\frac{\sigma_{r}-\sigma_{\theta }}{\rho }=0.$$

One can eliminate the derivative ∂ψ/∂ρ in this equation using (17). Then, Equation (35) becomes

(36) $$\frac{\partial \sigma_{r}}{\partial \psi }+\frac{2\left(\sigma_{r}-\sigma_{\theta }\right)\cos\psi }{\left(\sqrt{3}\cos\psi -\sin\psi \right)}=0.$$

Using (33) and (34), Equation (36) can be transformed into

(37) $$\cos\gamma \frac{\partial \gamma }{\partial \psi }-\frac{\left(\sin\gamma -\sqrt{3}\cos\gamma \right)\cos\psi }{\left(\sin\psi -\sqrt{3}\cos\psi \right)}+\frac{\sqrt{3}c}{2}\frac{\partial \left(\xi_{r}^{pf}-\xi_{z}^{pf}\right)}{\partial \psi }-\frac{\sqrt{3}c\cos\psi \left(\xi_{r}^{pf}-\xi_{\theta }^{pf}\right)}{\left(\sin\psi -\sqrt{3}\cos\psi \right)}=0.$$

Since $\sigma_{r}=0$ at ρ=a at the end of unloading, it follows from (33) and (34) that the boundary condition to Equation (37) is

(38) $$\gamma =\gamma_{a}\;\mathrm{for}\;\psi =\psi_{a}$$

where $\gamma_{a}$ is determined from

(39) $$\sin\gamma_{a}=\frac{\sqrt{3}c}{2}\left(\xi_{z}^{pf}-\xi_{r}^{pf}\right).$$

The forward plastic strains involved in the definitions of $\xi_{z}^{pf}$ and $\xi_{r}^{pf}$ are understood to be calculated at ρ=a. Equation (37) should be solved numerically. It is worthy of note that the dependence of the third and fourth terms of this equation on ψ is known from the solution at loading described in the previous section. Therefore, the solution of Equation (37) satisfying the boundary condition (38) supplies the dependence of γ on ψ in the range $a\leq \rho \leq \rho_{s}$.

The solution of (26) must satisfy the boundary condition (13). Therefore, ΔA=−ΔB and Equation (26) becomes

(40) $$\frac{\Delta \sigma_{r}}{\sigma_{0}}=\Delta A\left(\frac{1}{\rho^{2}}-1\right)\;\mathrm{and}\;\frac{\Delta \sigma_{\theta }}{\sigma_{0}}=-\Delta A\left(\frac{1}{\rho^{2}}+1\right).$$

This solution is valid in the region $\rho_{s}\leq \rho \leq 1$. The distribution of the residual stresses in the region $\rho_{s}\leq \rho \leq \rho_{c}$ is determined from (16) and (40) as

(41) $$\frac{\sigma_{r}^{res}}{\sigma_{0}}=\Delta A\left(\frac{1}{\rho^{2}}-1\right)-\frac{2\sin\psi }{\sqrt{3}}\;\mathrm{and}\;\frac{\sigma_{\theta }^{res}}{\sigma_{0}}=-\Delta A\left(\frac{1}{\rho^{2}}+1\right)-\frac{\sin\psi }{\sqrt{3}}-\cos\psi .$$

Here, one can eliminate ρ (or ψ) using (19). Both $\sigma_{r}^{res}$ and $\sigma_{\theta }^{res}$ must be continuous across the elastic/plastic boundary $\rho =\rho_{s}$. Then, it follows from (33), (34) and (41) that

(42) $$\begin{array}{c} \frac{2\sin\gamma_{s}}{\sqrt{3}}+c\left(\xi_{r}^{pf}-\xi_{z}^{pf}\right)=\Delta A\left(\frac{1}{\rho_{s}^{2}}-1\right)-\frac{2\sin\psi_{s}}{\sqrt{3}},\\ \frac{\sin\gamma_{s}}{\sqrt{3}}+\cos\gamma_{s}+c\left(\xi_{\theta }^{pf}-\xi_{z}^{pf}\right)=-\Delta A\left(\frac{1}{\rho_{s}^{2}}+1\right)-\frac{\sin\psi_{s}}{\sqrt{3}}-\cos\psi_{s}. \end{array}$$

The forward plastic strains involved in the definitions of $\xi_{z}^{pf}$, $\xi_{\theta }^{pf}$ and $\xi_{r}^{pf}$ are understood to be calculated at $\rho =\rho_{s}$. Additionally, $\psi_{s}$ and $\gamma_{s}$ are the values of ψ and γ at $\rho =\rho_{s}$, respectively. One can eliminate ΔA between the equations in (42) to arrive at

(43) $$\begin{array}{c} \left[\frac{2\sin\psi_{s}}{\sqrt{3}}+\frac{2\sin\gamma_{s}}{\sqrt{3}}+c\left(\xi_{r}^{pf}-\xi_{z}^{pf}\right)\right]\left(1+\rho_{s}^{2}\right)+\\ \left[\frac{\sin\psi_{s}}{\sqrt{3}}+\cos\psi_{s}+\frac{\sin\gamma_{s}}{\sqrt{3}}+\cos\gamma_{s}+c\left(\xi_{\theta }^{pf}-\xi_{z}^{pf}\right)\right]\left(1-\rho_{s}^{2}\right)=0. \end{array}$$

It follows from (19) that

(44) $$\rho_{s}=a\exp\left[\frac{\sqrt{3}}{2}\left(\psi_{s}-\psi_{a}\right)\right]\sqrt{\frac{\sin\left(\psi_{a}-\pi /3\right)}{\sin\left(\psi_{s}-\pi /3\right)}}.$$

Using (44), one can eliminate $\rho_{s}$ in (43). The solution of Equation (37) supplies the dependence of $\gamma_{s}$ on $\psi_{s}$. As a result, Equation (43) contains one unknown $\psi_{s}$. This resulting equation should be solved for $\psi_{s}$ numerically. Then, $\gamma_{s}$ is found from the solution of Equation (37) and $\rho_{s}$ from (44). The value of ΔA can be determined from any equation in (42). For example,

(45) $$\Delta A=\frac{\left[\frac{2\sin\psi_{s}}{\sqrt{3}}+\frac{2\sin\gamma_{s}}{\sqrt{3}}+c\left(\xi_{r}^{pf}-\xi_{z}^{pf}\right)\right]\rho_{s}^{2}}{\left(1-\rho_{s}^{2}\right)}.$$

This equation should be used for eliminating ΔA in (41).

The distribution of the residual stresses in the region $\rho_{c}\leq \rho \leq 1$ is determined from (14) and (40) as

(46) $$\frac{\sigma_{r}^{res}}{\sigma_{0}}=\left(A+\Delta A\right)\left(\frac{1}{\rho^{2}}-1\right)\;\mathrm{and}\;\frac{\sigma_{\theta }^{res}}{\sigma_{0}}=-\left(A+\Delta A\right)\left(\frac{1}{\rho^{2}}+1\right).$$

As before, ΔA in this equation should be eliminated by means of Equation (45).

The distribution of the residual stresses in the region $a\leq \rho \leq \rho_{s}$ is determined as follows. One can transform Equations (33) and (34) to

(47) $$\frac{\sigma_{r}^{res}}{\sigma_{0}}=\frac{2\sin\gamma }{\sqrt{3}}+c\left(\xi_{r}^{pf}-\xi_{z}^{pf}\right)\;\mathrm{and}\;\frac{\sigma_{\theta }^{res}}{\sigma_{0}}=\frac{\sin\gamma }{\sqrt{3}}+\cos\gamma +c\left(\xi_{\theta }^{pf}-\xi_{z}^{pf}\right).$$

In these equations, γ is a known function of ψ due to the solution of Equation (37). Then, (19) and (47) supply the dependence of the residual stresses on ρ in parametric form, with ρ being the parameter.

**Figure 3:**
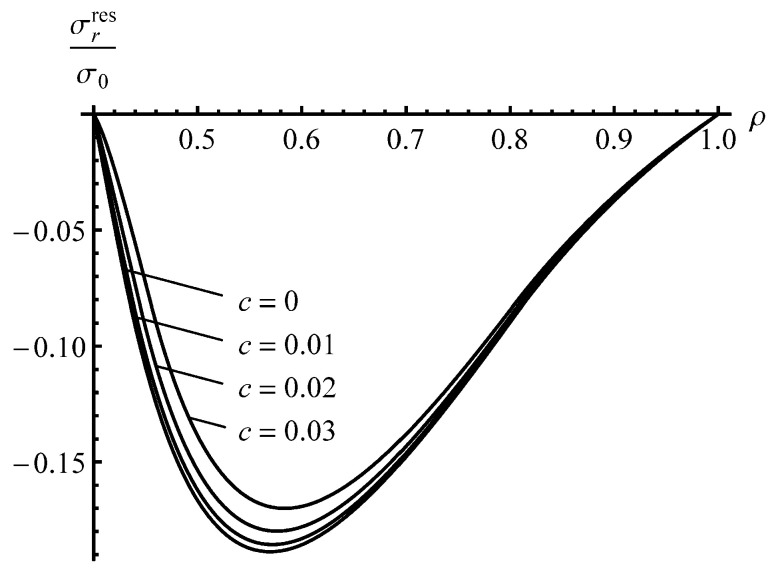
Distribution of the residual radial stress in an *a* = 0.4 cylinder.

**Figure 4:**
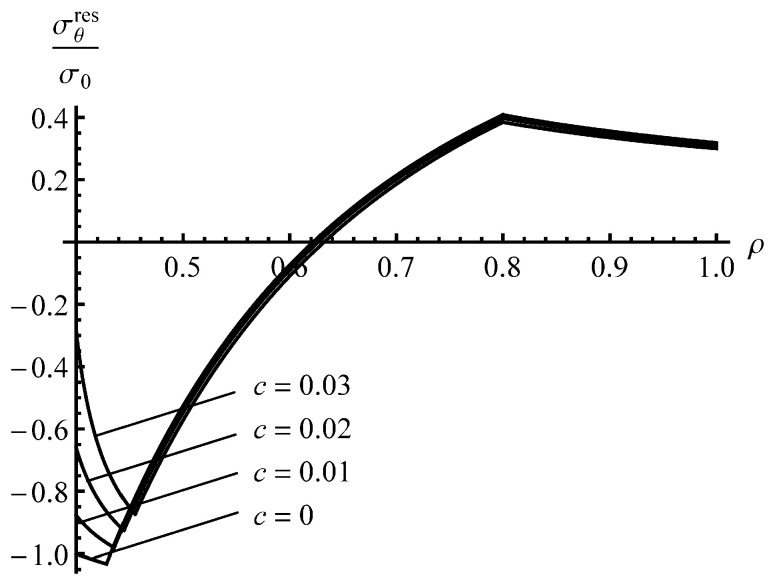
Distribution of the residual circumferential stress in an *a* = 0.4 cylinder.

**Figure 5:**
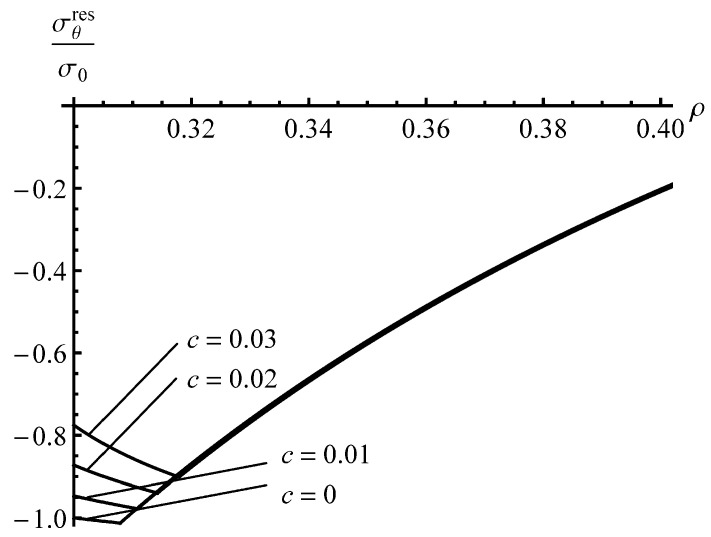
Distribution of the residual circumferential stress near the inner radius of an *a* = 0.3 cylinder.

The solution found is illustrated in Figure 3 and Figure 4 for an a=0.4 cylinder and several values of *c*. It has been assumed that ν=0.3. The special case, c=0, corresponds to the material that reveals no Bauschinger effect. The stage of loading ends when $\rho_{c}=0.8$. The corresponding value of the internal pressure is $p_{f}=0.97$ (approximately). Figure 3 displays the variation of the residual radial stress with the dimensionless radius. The effect of the *c*—value is not so significant. This is not surprising because the value of this stress at ρ=a and ρ=1 is controlled by the boundary conditions. Figure 5 shows the variation in the residual circumferential stress with the dimensionless radius. The effect of the *c*—value on this stress is significant in the vicinity of the inner radius where the magnitude of the circumferential stress is the most significant quantity in autofrettage technologies. It is seen from Figure 3 that an increase in the Bauschinger effect leads to a decrease in the value of $\left|\sigma_{\theta }^{res}\right|$ at the inner radius of the cylinder, which has a negative impact on its performance under service conditions.

To reveal an effect of *a* on the distribution of the residual stresses, the solution for an a=0.3 cylinder has been found assuming that $p_{f}=0.97$. The effect of *c*—value on the distribution of the residual radial stress is even smaller than that shown in Figure 3. Therefore, the distribution of this stress at a=0.3 is not illustrated. It is seen from Figure 4 that the effect of *c*—value on the distribution of the residual circumferential stress is negligible in the range $\rho_{s}\leq \rho \leq 1$. Therefore, Figure 4 shows the distribution of the residual circumferential stress near the inner radius of an a=0.3 cylinder. It is seen from this figure that the Bauschinger effect has a significant impact on this stress near the inner radius. Comparison of the distributions of the residual circumferential stress near the inner radius for the a=0.4 and a=0.3 cylinders (Figure 4 and Figure 5) shows that the magnitude of this stress at ρ=a is sensitive to both *a* and *c* at the same value of $p_{f}$. It is worthy of note that there is no need to solve the boundary value problem at unloading to find the value of $\sigma_{\theta }^{res}$ at ρ=a.

It follows from (11) and (13) that $\sigma_{r}^{res}=0$ at ρ=a. Then, the yield criterion (5) at ρ=a becomes

(48) $$\frac{3}{2}\left[{\left(\frac{\sigma_{\theta }^{res}}{3\sigma_{0}}+c\xi_{r}^{pf}\right)}^{2}+{\left(\frac{2\sigma_{\theta }^{res}}{3\sigma_{0}}-c\xi_{\theta }^{pf}\right)}^{2}+{\left(\frac{\sigma_{\theta }^{res}}{3\sigma_{0}}+c\xi_{z}^{pf}\right)}^{2}\right]=1.$$

The forward plastic strains involved in the definitions of $\xi_{z}^{pf}$, $\xi_{\theta }^{pf}$ and $\xi_{r}^{pf}$ are understood to be calculated at ρ=a. Equation (48) is a quadratic equation for $\sigma_{\theta }^{res}/\sigma_{0}$. The solution of this equation, which is in agreement with the physical meaning of $\sigma_{\theta }^{res}$, is

(49) $$\frac{\sigma_{\theta }^{res}}{\sigma_{0}}=\frac{3c\xi_{\theta }^{pf}-\sqrt{3c^{2}{\left(\xi_{\theta }^{pf}\right)}^{2}-6c^{2}\left[{\left(\xi_{r}^{pf}\right)}^{2}+{\left(\xi_{z}^{pf}\right)}^{2}\right]+4}}{2}.$$

**Figure 6:**
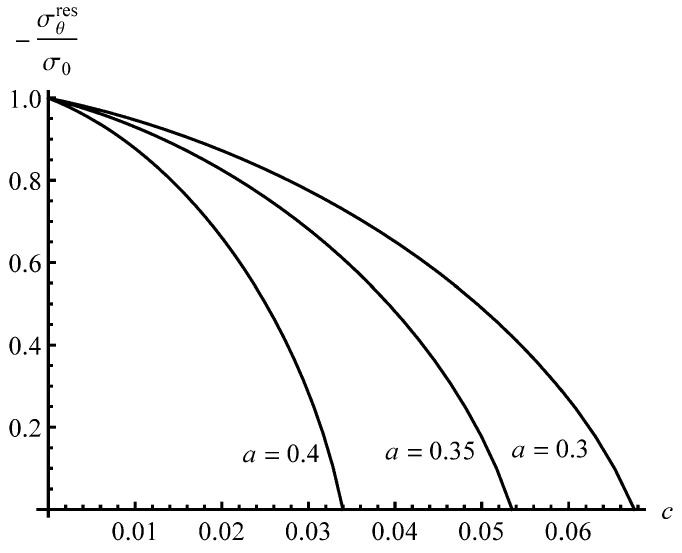
Effect of *c*—value on the residual circumferential stress at the inner radius of the cylinder for several values of *a*.

The equation $\xi_{r}^{pf}+\xi_{\theta }^{pf}+\xi_{z}^{pf}=0$, which follows from the equation $\varepsilon_{r}^{pf}+\varepsilon_{\theta }^{pf}+\varepsilon_{z}^{pf}=0$, has been used to derive (49). Using (49), the residual circumferential stress has been calculated at ρ=a to show the sensitivity of this stress to both *a* and *c*. Figure 6 illustrates this solution.

## 5. Conclusions

A new theory of the autofrettage process of a long open-ended cylinder has been developed. The theory accounts for the Bauschinger effect according to the material model proposed in [9]. This model takes into account some typical features in the behavior of high-strength steel. 

The solution is semi-analytic. If the solution at the end of loading is available, then numerical techniques are only necessary to solve the ordinary differential Equation (37) and several transcendental equations. Therefore, the solution can be used as a benchmark problem for verifying numerical codes, which is a necessary step before using such codes [22,23].

It has been shown that the impact of the Bauschinger effect on the distribution of the residual stresses outside the reversed plastic region is not significant, but is quite substantial, on the magnitude of the residual circumferential stress near the inner radius of the cylinder (Figure 4 and Figure 5). The latter is very important for autofrettage technologies. Therefore, it is essential to account for the Bauschinger effect in analyses of the autofrettage process, even if this effect is not so significant.

The possibility of finding rather a simple solution, which is very important for structural design, arises from using the model illustrated in Figure 2. This model is a result of the approximation of standard mechanical tests [1,2,3,4,24]. Therefore, its justification from the viewpoint of material scientists is desirable. From this point of view, the Bauschinger effect is generally explained by internal stresses that assist the motion of dislocations in the reverse direction. The dislocation pile-up and tangle are the main sources of such internal stresses. Another approach is to interpret the Bauschinger effect by the composite model in which the inhomogeneous internal stress state is attributed to a modulus difference effect within the microstructure [25]. The latter fits better to explain the phenomenon of insignificant work hardening and yet a significant Bauschinger effect that occurs in high-strength steels.

The method of solution used in the present paper can be extended to other autofrettage technologies such as rotational and thermal autofrettage [12,13]. In particular, the corresponding solutions at loading are already available [26,27]. The combination of these solutions and the method developed will be the subject of a subsequent investigation.
